# SWATH-MS proteomic data set of *Campylobacter jejuni* and *Campylobacter coli* exposed to the ionophore PBT2

**DOI:** 10.1128/mra.00789-26

**Published:** 2026-08-28

**Authors:** Huiting Chen, Ibrahim M. El-Deeb, Mark von Itzstein, Michael P. Jennings, Freda E.-C. Jen

**Affiliations:** 1 Institute for Biomedicine and Glycomics, Griffith University 5723, Gold Coast, Australia; Fluxus Inc., Sunnyvale, California, USA

**Keywords:** *Campylobacter jejuni*, *Campylobacter coli*, PBT2, ionophore, antimicrobial resistance

## Abstract

*Campylobacter jejuni* and *Campylobacter coli* are major foodborne pathogens. We announce the deposition of Sequential Window Acquisition of All Theoretical Mass Spectra (SWATH-MS) proteomic data sets from PBT2-treated and untreated cultures. These data sets provide a resource for investigating the proteomic response of *Campylobacter* spp. to the antimicrobial ionophore PBT2.

## ANNOUNCEMENT

*Campylobacter* spp. are gram-negative bacteria and a leading cause of bacterial gastroenteritis worldwide (1). *Campylobacter jejuni* and *Campylobacter coli* are the most clinically relevant species and are commonly transmitted through contaminated poultry, water, or unpasteurized milk (1). PBT2 is a zinc ionophore that was originally developed for neurodegenerative diseases (2, 3). Recent studies have demonstrated its antibacterial activity against gram-positive and gram-negative bacteria through disruption of intracellular metal homeostasis (4–14). Here, we present Sequential Window Acquisition of All Theoretical Mass Spectra (SWATH-MS)-based quantitative proteomics data sets (15) generated from *C. coli* and *C. jejuni* cultures exposed to PBT2 at various concentrations and sampled at defined time points.

*C. jejuni* ATCC 33560 and *C. coli* ATCC BAA-1061 (RM2228) were cultured on Mueller-Hinton (MH) agar supplemented with 10% defibrinated horse blood at 42°C for 18 h under microaerobic conditions. Colonies were inoculated in MH broth (BD Difco) supplemented with 5% defibrinated horse blood at OD_600_ = 0.001. *C. jejuni* cultures were harvested 12 h after exposure to 0.625 or 0.313 mg/L PBT2 and 24 h after exposure at 0.313 mg/L. *C. coli* cultures were harvested at 4 h after exposure to PBT2 at concentrations of 1.25 and 0.313 mg/L and 24 h after exposure at 0.313 mg/L. Untreated controls were collected at each time point. All experiments were performed in biological triplicate.

Sample preparation for proteomic analysis was performed as previously described (12). Briefly, cells were harvested by centrifugation at 4,500 rpm for 5 min at 4°C and washed three times with PBS. Cells were lysed with 8 M urea in 50 mM ammonium bicarbonate and sonicated for 5 min. Samples were centrifuged at 18,000 × *g* for 10 min at 4°C to remove debris. Supernatants were collected, and DTT was added (5 mM final) and incubated at 56°C for 30 min. Iodoacetamide (IAA) was added to a final concentration of 25 mM and incubated for 30 min at room temperature in the dark. DTT was again added to quench excess IAA. Samples were diluted with 50 mM ammonium bicarbonate and incubated with trypsin at 37°C overnight. Peptides were desalted using C18 Zip Tip (Millipore) and eluted with 0.1% formic acid in 70% acetonitrile (ACN). The peptides were dried using SpeedVac and resuspended in 0.1% formic acid for liquid chromatography-tandem mass spectrometry (LC-MS/MS) analysis.

SWATH-MS-based proteomics analysis was performed as previously described (12, 16). Liquid chromatography was performed on a M-Class UPLC system (Waters) coupled to a ZenoTOF 7600 (Sciex). Peptides were separated in reversed-phase using a Waters NanoEase HSS T3 column (100 Å, 1.8 µm, 300 µm × 150 mm) at 40°C with a flow rate of 5 µL/min. The LC gradient was set as follows: 0–0.6 min = 5% buffer B, 0.6–22 min = 5%–35%, 22–23 min = 35%–90%, held at 90% buffer B for 3 min followed by re-equilibration for 4 min with buffer A before next injection. Buffer A: 0.1% formic acid in water; buffer B: 0.1% formic acid in ACN. A time-of-flight mass spectrometry scan was performed across an *m*/*z* precursor mass range of 400–1,500 with an accumulation time of 0.1 s. MS/MS spectra were acquired using variable windows spanning *m*/*z* 399.5–750.5, with an accumulation time of 0.013 s per window. Fragment ions were collected across *m*/*z* 140–1,750. Zeno pulsing was enabled with a 100,000 cps threshold, and dynamic collision energy was applied throughout. The ion source gas 1 and 2 were set to 20 and 15 psi, respectively, curtain gas to 35, collisionally activated dissociation (CAD) gas to 7, and source temperature to 150°C; spray voltage was set to 5,000 V. Peptides were identified using DIA-NN (version 1.8, Sciex) (17) against the predicted proteomes of *C. jejuni* ATCC 33560 and *C. coli* RM2228. The protein sequence databases were obtained from the National Center for Biotechnology Information annotations associated with RefSeq assembly GCF_000254515.1 (GCF_000254515.1_ASM25451v1_protein.faa) and GenBank assembly GCA_000167415.1 (GCA_000167415.1_ASM16741v1_protein.faa), respectively, and were downloaded on 9 October 2024. Peptide quantification was performed using PeakView (version 2.2, Sciex), and protein abundances were filtered to a 1% false discovery rate threshold. Differential analyses were performed using MSstats in R (18, 19). Differentially abundant proteins were defined using the threshold adj. *P* < 0.05, Fold Change > 2.

We have identified a total of 1,224 and 802 proteins in *C. jejuni* and *C. coli*, respectively. Importantly, global proteomic profiles were altered following PBT2 treatment. Proteins associated with translation, respiration, cell envelope integrity, motility, and metal homeostasis exhibited altered abundance following PBT2 exposure. These data sets provide a resource for investigating the proteomic response of *Campylobacter* spp. to ionophore PBT2.

## Data Availability

The mass spectrometry proteomics data have been deposited to the ProteomeXchange (20) Consortium via the PRIDE (21) partner repository with the data set identifier PXD080115.
